# Gleanings in Science and Medicine

**Published:** 1893-04-15

**Authors:** 


### Gleanings in Science and Medicine.

A decree for the protection of frogs has lately been issued by the King of the Belgians. This law declares that no Belgian subject may either capture or destroy frogs or expose them for sale, unless they are bred for the French market, as is the case in various tracts of land in Belgium. And one other exception to the rule thus laid down for the preservation of frogs is made in favour of such persons as require the animals for the purpose of scientific research.

An interesting exhibition concerning fish and fish culture will be opened at Truro in July. The aim of this exhibition will be the development of pisciculture in the United Kingdom, and the propagation of the best known methods in fluvial and maritime fishing. The originator of the scheme is Mr. Lawrence Hamilton, who is anxious that a Royal Commission should be called to inquire into all questions relative to the preservation of fish, from the triple standpoint of food, commerce, and cheap selling. In China, fish constitutes at once the most abundant and most economical food within the reach of all classes, and Mr. Hamilton is devoting his energies to trying to effect a like state of things in his own country.

A weekly contemporary gives a curious example of how dominant in their natures is the sense of smell among the New Zealanders; it permeates their very existence, and finds expression in their song. From their infancy the children are encouraged to love sweet scents; indeed, many of our best ones come from their island home. A Maori mother lulls her babe to sleep with words curiously characteristic of her nationality, with lullabies wherein melody and rhyme are made subservient to the theme, which is ever illustrative of sweet perfumes, a very favourite one being thus translated:—

My little neck-satchel of sweet-scented moss,
My little neck-satchel of fragrant fora,
My little neck-satchel of odoriferous gum,
My sweet-smelling neck-locket of sharp-pointed Taramea.

The manufacture of Japanese trays is not confined to Birmingham, as we constantly hear it asserted. Whole families in Japan depend for their livelihood on the making of these trays, not on a wholesale manufacture by machinery, but through a slow and laborious process by the hand. Indeed, the better trays, those which are inlaid with mother-of-pearl and ivory, are not turned out daily by the gross, but occupy a family often as much as six months in their construction; in this work each member plays his own appointed part, there being several processes for the completion of the tray. The lashing together of the wood, the glutinous preparation covering it, the designing, and the inlaying. It is, indeed, almost surprising that the result of so much labour can be purchased at so comparatively low a price.

Amongst the numerous trades which the Parisian capital boasts of is the somewhat unique business carried on by over 100 butchers who deal exclusively in horseflesh. This commodity is cheaper than ordinary meat, and finds a ready sale, in fact, its supply is not entirely limited to the tables of the poor, but is served up, under the name of "beef" and "mutton" in far more luxurious quarters. We feel much tempted to ask what course of circumstances renders horseflesh of less value in the market than that of oxen and sheep, but we are not told the secrets of the trade on which 150 butchers thrive, and we can only imagine that the reduction in the price of this spurious "beef" and "mutton" is due to condition of the animal, before it appears in the slaughter-house, which it is, perhaps, to as well conceal from the demolisher.

In the Valley of the Thames, and especially at Richmond, rumour reports the appearance lately of earth-worms which are luminous. Naturalists tell us that the presence of those phosphorescent annelids is not uncommon in our midst, but we can only say that our own experience has not taught us so. Earthworms have long been a pet subject of the scientist, and a curious fact has lately been brought to light in reference to the connection now asserted to exist between the small single-celled animals and the loathsome disease of "cancer." We must have all of us have noticed from time to time numbers of ordinary earth-worms lying on the surface of the earth, temptingly exposed to the eye of birds, and yet left untouched by them. Recent investigations have proved that the tail end of these worms are filled with animals closely resembling the same which are supposed to create the so-called "cancer" in the human form; and, which, in the case of the earth-worms thus investigated, have doubtless also been the cause of death.

Miss Mary Garrett, we hear, is not contented with the manner in which her munificent contribution is being carried out at the John Hopkins University. Her subscription completed the sum of money required to open a medical school where women are to be admitted on equal terms with men, and it is to be hoped that so great a benefactress to her sex will find that the arrangements will satisfactorily mend themselves more to her liking. America has ever been instrumental in the encouragement of female physicians in its midst. The John Hopkins School of Medicine is only an extension of the sympathy which our transatlantic cousins have already shown to women who would study science in a practical manner. A new monthly journal devoted to the interests of the female side of the profession has lately made its appearance, hailing from Toledo, O., and describing itself as *The Woman's Medical Journal*. The arguments which arise in our mind as to the probability of success attending a new venture on lines already somewhat overrun are masterfully anticipated in the preface of the first number of this magazine. We can only wish success and prosperity to an editor who thus faces his difficulties in so spirited a manner.

